# Expression of 22 serotonin-related genes in rat brain after sub-acute serotonin depletion or reuptake inhibition

**DOI:** 10.1017/neu.2020.9

**Published:** 2020-06

**Authors:** Jakob Näslund, Erik Studer, Staffan Nilsson, Elias Eriksson

**Affiliations:** 1 Department of Pharmacology, Institute of Neuroscience and Physiology at the Sahlgrenska Academy, University of Gothenburg, Gothenburg, Sweden; 2 Division of Applied Mathematics and Statistics, Department of Mathematical Sciences, Chalmers University of Technology, Gothenburg, Sweden

**Keywords:** Serotonin, SSRI, amygdale, hippocampus, prefrontal cortex

## Abstract

**Objective:**

Although the assessment of expression of serotonin-related genes in experimental animals has become a common strategy to shed light on variations in brain serotonergic function, it remains largely unknown to what extent the manipulation of serotonin levels causes detectable changes in gene expression. We therefore chose to investigate how sub-acute depletion or elevation of brain serotonin influences the expression of a number of serotonin-related genes in six brain areas.

**Methods:**

Male Wistar rats were administered a serotonin synthesis inhibitor, *para*-chlorophenylalanine (*p*-CPA), or a serotonin reuptake inhibitor, paroxetine, for 3 days and then sacrificed. The expression of a number of serotonin-related genes in the raphe nuclei, hypothalamus, amygdala, striatum, hippocampus and prefrontal cortex was investigated using real-time quantitative PCR (rt-qPCR).

**Results:**

While most of the studied genes were uninfluenced by paroxetine treatment, we could observe a robust downregulation of tryptophan hydroxylase-2 in the brain region where the serotonergic cell bodies reside, that is, the raphe nuclei. *p*-CPA induced a significant increase in the expression of *Htr1b* and *Htr2a* in amygdala and of *Htr2c* in the striatum and a marked reduction in the expression of *Htr6* in prefrontal cortex; it also enhanced the expression of the brain-derived neurotrophic factor (*Bdnf*) in raphe and hippocampus.

**Conclusion:**

With some notable exceptions, the expression of most of the studied genes is left unchanged by short-term modulation of extracellular levels of serotonin.

## Significant outcomes

- Even drastic alterations of extracellular serotonin levels translate into comparatively minor changes in serotonergic gene expression.
- While transcript levels of serotonergic genes in the raphe region and areas such as amygdala and striatum were clearly affected by manipulations of serotonin levels; small or no changes could be observed in the hypothalamus and prefrontal cortex.
- Short-term SSRI treatment is associated with downregulation of genes encoding enzymes regulating serotonin synthesis in the raphe nuclei (*Tph2*, *Ddc*).

## Limitations

- The dissection techniques employed unfortunately preclude analysis of potentially important effects in sub-areas, such as differences in expression patterns between the dorsal and median raphe nuclei.
- Although analysis of behavioural correlates of observed effects on gene expression could have been potentially informing, this is not possible as no such tests were performed.
- As with all gene expression studies, the functional relevance (e.g. the impact on protein levels and function) is somewhat unclear.

## Introduction

The assumption that brain serotonin plays a key role in the regulation of various aspects of human behaviour, and is the target for important psychoactive drugs, has inspired numerous attempts to measure brain serotonergic activity in experimental animals. To this end, a variety of techniques has been applied, among which assessment of the expression of serotonin-related genes, not least because of its convenience, has become one of the most commonly used. Thus, analysis of the expression of genes encoding serotonin-related proteins has, for example, been applied for addressing how serotonergic transmission may be influenced by factors such as drugs (Barbon *et al.*, 2011; Yamamura *et al.*, 2011; McQuade *et al.*, 2004), electroconvulsive treatment (Shen *et al.*, 2003), hormones (McQueen *et al.*, 1999; Donner & Handa, 2009), maternal separation (Gardner *et al.*, 2009), stress (Bethea *et al.*, 2013) and social defeat (Boyarskikh *et al.*, 2013).

While the underlying assumption for this strategy has been that messenger RNA (mRNA) levels to some extent may reflect the levels of the corresponding protein, and that regulation of gene expression may be one important route for factors exerting long- or short-term influences on brain serotonergic activity, it remains to be clarified to what extent up- and downregulation of gene expression are indeed important mechanisms for inducing transient fluctuations and/or stable alterations in the status of a certain transmitter, for example, for the purpose of maintaining homeostasis. For example, while studies such as those cited above indicate that the expression of serotonin-related genes is indeed influenced by various external influences, it has been shown that knockout of the gene encoding the serotonin-synthesising enzyme tryptophan hydroxylase 2 (*Tph2*), leading to marked depletion of brain serotonin levels, did not influence the expression of any of a large number of serotonin-related genes that were assessed (Kriegebaum *et al.*, 2010). Thus, to facilitate the interpretation of studies using mRNA assessment to reflect the status of brain serotonergic transmission, it is important to increase the insight into how responsive the expression of various serotonin-related genes is to interventions known to influence extracellular serotonin levels.

In this vein, the present study was undertaken to evaluate the possible influence of short-term alterations in synaptic levels of serotonin on a large number of serotonin-related genes, both in the raphe nuclei, where the serotonergic cell bodies reside, and in various terminal regions; the hypothesis being that such changes would induce rapid adaptive responses in some but not all of the studied genes. To this end, one group of animals was injected for 3 days with the selective serotonin reuptake inhibitor (SSRI) paroxetine, that is, a drug that is likely to cause at least a moderate increase in extrasynaptic levels of serotonin in at least some of the studied terminal regions (see refs in Fuller, 1994) and, at the same time, to dampen the electrical activity of the serotonergic cell bodies in the raphe region (as the result of an autoreceptor-mediated feedback) (Hajós *et al.*, 1995). Another group of rats was subjected to 3 days of treatment with the serotonin synthesis inhibitor, *para*-chlorophenylalanine (*p*-CPA), in a regimen known to cause a depletion of tissue serotonin exceeding 95% (Miczek *et al.*, 1975; Näslund *et al.*, 2013). Supporting that the applied treatments do in fact produce a significant alteration of serotonergic output, they are known to elicit reciprocal behavioural effects that are likely to be the result of such an influence; thus, while *p*-CPA enhances aggression (Miczek *et al.*, 1975) and sexual activity (Tagliamonte *et al.*, 1969) and reduces anxiety-like behaviour (as assessed, e.g. using the elevated plus maze; Treit *et al.*, 1993; Näslund *et al.*, 2015), acute administration of SSRIs leads to the opposite effects (Griebel *et al.*, 1994; Vega Matuszcyk *et al.*, 1998; Ho *et al.*, 2001).

### Aims of the study

We aimed to investigate, by way of measurement of transcript levels of a comprehensive set of serotonergic genes, the responsiveness of the serotonin system to short-term manipulation of synaptic serotonin levels. We also desired to characterise the effects of such interventions *per se* and to compare these results with earlier work by other groups.

## Experimental procedures

### Study design and protocol

Thirty-three male Wistar rats, aged 9–10 weeks at arrival, were kept in an animal facility for 5 weeks, whereupon they were randomly divided into three equal groups and administered paroxetine, *p*-CPA or saline for 3 days. On the morning of the 4th day, they were given a final injection and sacrificed 2 h later. Brains were immediately extracted and dissected on dry ice.

In five regions receiving substantial serotonergic innervation, that is, amygdala, hippocampus, striatum, hypothalamus and prefrontal cortex, we assessed the genes encoding *i)* nine different serotonergic receptor subtypes, 5HT1A (*Htr1a*), 5HT1B (*Htr1b*), 5-HT1D (*Htr1d*), 5-HT2A (*Htr2a*), 5-HT2C (*Htr2*c), 5-HT3A (*Htr3a*), 5-HT4 (*Htr4*), 5-HT6 (*Htr6*) and 5-HT7 (*Htr7*); *ii)* the two subtypes of the monoamine metabolising enzyme, monoamine oxidases A and B (*Maoa* and *Maob*); *iii)* brain-derived neurotrophic factor (*Bdnf*), which is a protein known to exert an important impact on serotonergic transmission (Rumajogee *et al.*, 2005); *iv)* the BDNF receptor (*Ntrk1*); and *v)* P11 (*S100a10*), which is another protein attributed to important interactions with brain serotonergic neurons (Anisman *et al.*, 2008; gene names within parentheses). In the raphe nuclei, where the serotonergic cell bodies reside, we studied a number of genes known to be expressed by serotonergic neurons and/or suggested to interact with these, such as those encoding *i)* the three serotonergic autoreceptors, that is, *Htr1a*, *Htr1b* and *Htr1d*; *ii)* the enzymes involved in the synthesis of serotonin, that is, one of the two isoforms of tryptophan hydroxylase (*Tph2*) and amino acid-decarboxylase (*Ddc*); *iii)* the serotonin transporter (*Slc6a4*); *iv)* the monoamine vesicular transporter (*Slc18a2*); *v*) *Maoa* and *Maob*; *vi)* three transcription factors expressed by serotonergic neurons and of importance for the development (and possibly maintenance) of serotonergic neurons and expressed by these: GATA-2 (*Gata2*; Craven *et al.*, 2004), MASH-1 (*Ascl1*; Pattyn *et al.*, 2004) and PET*-1* (*Fev*) (Liu *et al.*, 2010); *vii*) *Bdnf*; *viii*) *Ntrk2*; and *ix)* *S100a10*. Finally, in order to shed further light on the possible differences between different regions with respect to how strongly they were influenced by the two interventions, the expression of the immediate-early gene *c*-*Fos* was assessed both in raphe and in the different terminal regions.

### Animals

Animals were obtained from Taconic (Ejby, Denmark) and housed with a 12-h light/dark cycle (lights on at 06:00 a.m.) and with standard chow and water available *ad libitum*. All procedures were carried out in accordance with national and European legislation and with the approval of the local ethics committee and in accordance with the institutional guidelines.

### Drug treatment

The *p*-CPA (Sigma-Aldrich, St Louis, MO, USA) was dissolved in 0.9% saline and administered intraperitoneally as one injection of 300 mg/kg per day for 3 days, with the last injection being given roughly 24 h before sacrifice. Paroxetine hydrochloride (Jai Radhe Chemicals, Ahmedabad, India) was dissolved in 0.9% saline and administered subcutaneously at a dose of 10 mg/kg 2 times per day. All animals were given two injections daily 10–12 h apart; the paroxetine group was given paroxetine 10 mg/kg at both occasions, the *p-*CPA group was given *p-*CPA (300 mg/kg) in the first injection and saline in the next and the saline group was given saline at both occasions. On the 4th day, the paroxetine group was administered a last dose of paroxetine 2 h before sacrifice, while the other two groups received saline.

### Dissection

Brains were extracted immediately after decapitation. The extended amygdala, hippocampus, striatum, hypothalamus, prefrontal cortex and raphe nuclei were dissected out for gene expression analysis. Brain tissue samples were immediately frozen on dry ice and stored at −80°C.

### Gene expression

Individual samples of brain tissue were homogenised in Qiazol (Qiagen, Hilden, Germany) using a TissueLyzer (Qiagen). Total RNA was extracted with an RNeasy Lipid Tissue Mini Kit (Qiagen) using a QiaCube (Qiagen). RNA quantities were determined, and quality assessed, by means of spectrophotometric measurements (Nanodrop 1000; Thermo Scientific, Wilmington, DE, USA). For complementary DNA (cDNA) synthesis, 4000 ng of total RNA was reversely transcribed using random hexamers (Applied Biosystems, Sundbyberg, Sweden) and Superscript III reverse transcriptase (Invitrogen Life Technologies, Paisley, UK) according to the manufacturer’s description. Recombinant RNaseout® Ribonuclease Inhibitor (Invitrogen) was added to prevent RNase-mediated degradation. All cDNA reactions were run in duplicate and the products pooled for further analysis.

Real-time qPCR was performed by means of TaqMan® Custom Arrays using TaqMan probe and primer sets for target genes and reference genes chosen from an online catalogue (Applied Biosystems). Two separate cards were used: one to investigate the raphe region and one to investigate the various target areas. Names and assay numbers for genes investigated are shown in Supplementary Table 1. The sets were factory loaded into the 384 wells of the TaqMan® Array, each port being loaded with cDNA corresponding to 500 ng total RNA combined with nuclease-free water and 50 µl TaqMan® Gene Expression Master Mix (Applied Biosystems) to a final volume of 100 µl. The TaqMan® Arrays were analysed using the 7900 HT system with a TaqMan Array Upgrade (Applied Biosystems). Thermal cycling conditions were 50°C for 2 min and 94.5°C for 10 min, followed by 40 cycles of 97°C for 30 s and 59.7°C for 1 min.

All reactions were run in duplicate, and reactions were excluded if the Cycle threshold (CT) values of the duplicates differed by more than 5%. By means of the NormFinder algorithm (http://moma.dk/normfinder-software), a combination of *Hmbs* and *Ppia* was found to display the highest stability among the four reference genes in all areas examined and was therefore used to normalise the expression levels. Gene expression values were calculated based on the ΔΔ*C*_t_ method (Livak & Schmittgen, 2001). Table 1 provides the relative expression of each gene; ΔΔC_t_ values for each gene are given in Supplementary Table 2.

**Table 1.** Gene expression effects of short-term manipulation of synaptic 5-HT levels

	Amygdala		Hippocampus		Striatum		Hypothalamus		Prefrontal cortex		Raphe	
	*p*-CPA	SSRI	*p*-CPA	SSRI	*p*-CPA	SSRI	*p*-CPA	SSRI	*p*-CPA	SSRI	*p*-CPA	SSRI
*Htr1a*	1.03	1.11	1.06	1.12*	0.83	0.83	0.99	0.96	1.05	1.18	0.89 (*p* = 0.01)	0.92
*Htr1b*	1.20***	1.05	1.01	0.89	1.20 (*p* = 0.05)	1.11	1.09	0.91	1.09	1.09	0.69	0.99
*Htr1d*	1.17	1.00	1.09	1.59*	1.18 (*p* = 0.08)	1.16 (*p* = 0.07)	0.90	1.06	0.88	1.25	0.89	0.89
*Htr2a*	1.18**	0.96	1.15	0.98	1.22 (*p* = 0.05)	0.87	1.05	0.81**	0.99	0.95		
*Htr2c*	0.91	1.10	1.06	0.94	1.53**	1.43**	0.93	0.98	1.00	0.86		
*Htr3a*	1.18*	1.15*	1.11	0.99	1.21 (*p* = 0.09)	1.26*	1.10	0.92	0.95	1.02		
*Htr4*	1.12*	1.20**	1.14	1.20	1.09	1.10	0.93 (*p* = 0.09)	0.99	1.05	1.09		
*Htr6*	1.00	1.03	0.84*	1.03	1.01	1.07	0.97	1.04	0.71***	0.92		
*Htr7*	1.08	1.10 (*p* = 0.09)	1.08	1.10	0.97	0.99	0.99	0.98	1.00	0.93		
*Maoa*	0.96	1.13*	0.96	1.12 (*p* = 0.7)	1.02	1.11 (*p* = 0.09)	0.92 (p = .08)	0.96	0.90	0.99	0.90 (*p* = 0.09)	1.04
*Maob*	1.13 (*p* = 0.1)	1.23**	1.13	1.14**	1.19*	1.18 (*p* = 0.05)	1.14	0.98	0.99	1.18	1.13*	0.96
*Bdnf*	1.23*	0.87 (*p* = 0.07)	1.46***	1.03	1.05	0.79	1.13	0.88	1.04	0.79	1.77**	0.85
*Ntrk2*	1.08	1.04	1.11	1.00	1.13*	1.07*	1.01	1.00	0.92	1.07	0.95	0.94
*S100a10*	1.11	1.13	1.08*	1.09 (*p* = 0.1)	0.79	0.84	1.06	0.94	1.12	0.84	0.97	0.98
*Slc6a4*	0.90	1.08	0.90	1.27	0.72 (*p* = 0.06)	1.35*	0.51*	0.76	0.65*	1.25	0.65*	0.71 (*p* = 0.08)
*Tph2*											0.82	0.56**
*Ddc*											0.80*	0.78*
*Slc18a2*											0.93	0.84 (*p* = 0.1)
*Fev*											0.76 (*p* = 0.07)	0.66*
*Gata2*											0.84	0.91
*Ascl1*											0.87 (*p* = 0.07)	1.10
*Fos*	1.12	1.23	1.57***	1.23	1.06	1.53 (*p* = 0.07)	1.26 (*p* = 0.1)	1.33*	0.80	1.36	1.45***	1.13

Treatment effects of short-term treatment with *p*-CPA or paroxetine. Numbers indicate expression levels relative to the saline group, with asterisks (*) indicating level of significance for the comparison.

*n* = 10–11 (*Slc6a4* in amygdala = 9)

*p < 0.05, **p < 0.01, ***p < 0.001.

### Statistical analyses

Student’s *t*-tests were used to test for treatment effects as compared to placebo; we chose not to use an analysis of variance-based approach as we did not aim to investigate the possible differences between SSRI- and *p*-CPA-treated animals. As this was a hypothesis-driven study, where some of the observed genes were expected to change in a certain direction, whereas for others there was no *a priori* hypothesis, the results are presented without any correction for multiple testing; nevertheless, permutation analyses were performed and are included in the supplementary online information (Supplementary Table 3). SPSS for Mac version 21 (IBM, Chicago, IL, USA) was used for all statistical procedures, except for the permutation analysis, where R (R Core team, Vienna, Austria) was employed.

## Results

### *para*-Chlorophenylalanine

*Htr1b*, *Htr2a* and *Htr3a* were upregulated in the amygdala, while *Htr2c* was upregulated in the striatum. *Htr4* was upregulated in the amygdala, while *Htr6* was downregulated in hippocampus and prefrontal cortex. *Maob* was upregulated in the striatum and raphe. *Bdnf* was upregulated in the amygdala, hippocampus and raphe, while *Ntrk2* was upregulated in the striatum. *Slc6a4* was downregulated in the hypothalamus, prefrontal cortex and raphe. *S100a10* was upregulated in the hippocampus. *Ddc* was downregulated in the raphe. *Fos* was upregulated in the hippocampus and raphe (Table 1). Nine of the observed effects survived correction for multiple comparisons by means of permutation analyses with subsequent area-by-area Holm-Bonferroni correction: *Htr1b* and *Htr2a* in the amygdala; *Bdnf* in the hippocampus; *Htr2c* in the striatum; *Htr4* and *Slc6a4* in the prefrontal cortex; and *Bdnf,* *Slc6a4* and *Ddc* in the raphe (Supplementary Table 3).

### Paroxetine

*Htr1a* and *Htr1d* were upregulated in the hippocampus. *Htr2a* was downregulated in the hypothalamus. *Htr2c* and *Htr3a* were upregulated in the striatum, and the latter gene was also upregulated in the amygdala. *Htr4* was upregulated in the amygdala. *Maoa* was upregulated in the amygdala, while *Maob* was upregulated in amygdala and hippocampus. *Ntrk2* and *Slc6a4* were upregulated in the striatum. Three genes were significantly downregulated by paroxetine in the raphe region: *Tph2*, *Ddc* and *Fev*. *Fos* was upregulated in hypothalamus (Table 1). None of the effects except for the downregulation of *Tph2* in the raphe survived correction for multiple comparisons (Supplementary Table 3).

## Discussion

One major conclusion of this study is that both paroxetine and *p*-CPA left the expression of most of the studied genes unaffected, the major exceptions being discussed below. It may hence be concluded either that short-term changes in extracellular serotonin levels and/or serotonergic cell firing, elicit an adaptive modulation of the formation of only a minority of the proteins in question, or that such adaptations are not easily captured using conventional assessment of mRNA expression. Of note is, for example, that the expression of the gene encoding the 5HT1A receptor (Popova & Naumenko, 2013), which is regarded as one of the most important mediators of the effects of serotonin on postsynaptic neurons, and which also serves as an autoreceptor at serotonergic cell bodies, was not influenced by any of the two treatments.

The observation that serotonin depletion induced a marked upregulation of *Bdnf* both in the raphe region and in the hippocampus is in agreement with an earlier study (Zetterström *et al.*, 1999) as well as with a report on *Tph2* knock-out mice (Migliarini *et al.*, 2012). The lack of effect of short-term administration of paroxetine on *Bdnf*, both in raphe and in the terminal regions, is also in agreement with the literature, where acute and sub-acute SSRI treatment has been reported to exert either modest down-regulating or no effects on the expression of this gene (Nibuya *et al.*, 1995; Zetterström *et al.*, 1999; Mannari *et al.*, 2008). Long-term administration of SSRIs, on the other hand, is reported to increase *Bdnf* expression (Mannari *et al.*, 2008), that is, to exert an effect similar to that of acute serotonin depletion.

Apart from the effect on *Bdnf*, there were very minor effects of 72 h of serotonin depletion on the expression of serotonin-related genes in the raphe. This finding is indirectly in line with previous reports according to which *p*-CPA does not influence the firing rate of raphe serotonergic neurons, that is, that these neurons are not under tonic feedback inhibition by extracellular levels of serotonin (Aghajanian *et al.*, 1970; Chaput *et al.*, 1990). Likewise, blockade of 5-HT1A autoreceptors does not exert any effect on raphe firing when administered *per se* (Mundey *et al.*, 1994).

According to the traditional concept of denervation supersensitivity, an expected effect of serotonin depletion on serotonergic receptors would be one of compensatory upregulation. In line with this, we observed an increase in *Htr1b* and *Htr2a* expression in the amygdala and of *Htr2c* in the striatum, in *p*-CPA-treated animals, the latter finding being in line with a previous study (Compan *et al.*, 1998). In contrast, none of the other receptors were significantly upregulated by this treatment. While it cannot be excluded that also other receptors would have been upregulated had the depletion lasted for longer than 72 h, our results suggest that 5-HT1B and 5-HT2A receptors in the amygdala, and 5-HT2C receptors in the striatum, are particularly sensitive to changes in extracellular levels of serotonin.

With respect to the 5-HT6 receptor, the opposite effect of serotonin depletion was found; *p*-CPA thus induced a marked reduction in the expression of the gene encoding this receptor in prefrontal cortex and a somewhat less pronounced effect in the same direction in the hippocampus. A previous study found support for 5-HT6 receptors in the prefrontal cortex to exert an inhibitory influence on the local release of serotonin (Schechter *et al.*, 2008); so from the perspective of adaptive feedback, a drug-induced shortage of serotonin leading to a downregulation of a receptor inhibiting serotonin release bears some logic.

Previous studies have suggested that serotonin depletion, as obtained using reserpine for 3 days (Xiao *et al.*, 1999) or *p*-CPA for 1–2 (Rattray *et al.*, 1996) or 10 days (Linnet *et al.*, 1995), leads to downregulation of the expression of the gene encoding the serotonin transporter, that is, to a change in the expression of this gene, which would make sense from an adaptive point of view. Our observation of reduced levels of *Slc6a4* expression in several brain regions of serotonin-depleted animals is hence in line with earlier work.

It has since long been known that inhibition of serotonin reuptake, as the result of an autoreceptor-mediated feedback, elicits an immediate reduction in serotonergic cell firing (Gallager & Aghajanian, 1975; Hajós *et al.*, 1995) as well as a decrease in serotonin turnover (Carlsson *et al.*, 1969; Fuller & Wong, 1977). In line with these observations, raphe *Tph2* expression was markedly reduced in rats exposed to paroxetine for 3 days. The TPH2 enzyme being downregulated by SSRIs has been shown before, both by means of histochemical methods (MacGillivray *et al.*, 2010) and by means of assessment of mRNA expression (Dygalo *et al.*, 2006; Abumaria *et al.*, 2007; Shishkina *et al.*, 2007; Klomp *et al.*, 2014); however, a common view has been that such an effect requires weeks of treatment to be at hand (Dygalo *et al.*, 2006). In contrast, our results suggest that increased extracellular serotonin levels in the raphe region leads to a downregulation of *Tph2* that takes no more than 3 days to be manifest. In addition, it is of note that two other raphe genes that are (more or less) exclusively expressed by serotonergic neurons, and the expression of which hence might be expected to be influenced by the activity of the neurons, that is, *Ddc* and *Fev*, also displayed reduced expression in SSRI-treated animals, and that there was a tendency for *Slc6a4* in the same direction. A previous study showed reduced expression of *Slc6a4* after 7, but not 4, days of fluoxetine treatment (Neumaier *et al.*, 1996). With respect to the influence of paroxetine on serotonergic receptors, it is noteworthy that this was always one of up- rather than downregulation, with *Htr2a* in the hypothalamus being the sole exception.

It is noteworthy that the striatum, the hippocampus and the amygdala were the areas that appeared most influenced by sub-acute SSRI treatment and that this observation is also supported by the assessment of *c-Fos* activation (Beck, 1995; Torres *et al.*, 1998; Morelli *et al.*, 1999). Partly in line with this, previous studies using *in vivo* microdialysis have shown short-term administration of an SSRI to cause a marked increase in the extracellular levels of serotonin in the striatum (Kalén *et al.*, 1989), hippocampus (Sabol *et al.*, 1992; Bosker et al., 1995) and the amygdala (Sundblad & Eriksson, 1997; Bosker *et al.*, 2001); in contrast, the possible effect of the same treatment in prefrontal cortex, where we observed no effects on gene expression, appears less clear-cut and has remained a matter of controversy (Sarkissian *et al.*, 1990; Adell & Artigas, 1991; Gartside *et al.*, 1995).

To summarise, in order to facilitate the interpretation of studies assessing the expression of serotonin-related genes to gain insight into the status of brain serotonergic neurotransmission after various experimental interventions, we have explored to what extent gene expression is influenced by short-term changes in extracellular levels of serotonin and/or in serotonergic nerve activity. The results show some of the studied genes to be markedly influenced while most were not. Follow-up studies assessing the possible influence of similar manipulations of the extracellular levels of serotonin being in place for a more prolonged time period are warranted.

## Supplementary material

For supplementary material accompanying this paper visit https://doi.org/10.1017/neu.2020.9. click here to view supplementary material
