# Supplementary material for: Expression of 22 serotonin-related genes in rat brain after sub-acute serotonin depletion or reuptake inhibition
Source: Acta Neuropsychiatr. 2020 Feb 17;32(3):159–65. doi: 10.1017/neu.2020.9 (PMC7282867; doi:10.1017/neu.2020.9)
Supplement: Supplementary file 1 [file S0924270820000095sup001.docx]

**Supplementary online information to 'Expression of 22 serotonin-related genes in rat brain after subacute serotonin depletion or reuptake inhibition' by Näslund et al.**

*This document encompasses information regarding supplementary statistical analyses, and three supplementary tables with corresponding legends.*

*Methods*

Statistical analyses

The following analyses are complementary to those reported in the main paper and entail correction for multiple testing. Apart from corrected values for individual genes, p-values for a general effect on target areas themselves, were obtained.

All analyses were carried out separately for the raphe region and the target areas. Within the latter, the possible influence of treatment on a certain brain area was tested using the minimum *p*-value over all in investigated genes as test statistic and its significance evaluated by means of permutation analysis in R (100 000 permutations). The overall effect on a specific gene across all the investigated areas was assessed in an analogous way. In the next step, obtained *p*-values for each area were analyzed with respect to multiple inference with the Holm-Bonferroni method (Holm, 1979). Finally, *p*-values for individual genes within each area that had reached the level of significance in the earlier step were also corrected using the same technique.

*Results*

Paroxetine

Region-wise, uncorrected p-values suggest the influence of paroxetine to be larger in striatum (0.04), hypothalamus (0.05), amygdala (0.09) and hippocampus (0.1) than in frontal cortex (0.9); however, none of these p-values survived correction for multiple comparisons. Except for *Tph2* in raphe, no effects on individual genes remained significant after correction (Supplementary table 3).

*para*-Chlorophenylalanine

Region-wise, *p*-CPA exerted a significant effect on all brain areas but hypothalamus (*p*=0.5). After correction for multiple comparisons, the following effects of *p*-CPA on gene expression in terminal regions remained significant: an increase in the expression of *Htr1b* and *Htr2a* in the amygdala, an increase in the expression of *Htr2c* in the striatum, an increase in the expression of *Bdnf* in the hippocampus and raphe, and a decrease in the expression of *Htr6* in the cortex (Supplementary table 3).

*References*

**Holm, S.** (1979). A simple sequentially rejective multiple test procedure. *Scandinavian Journal of Statistics* **6(2)**, 65-70.

*Table legends*

Supplementary table 1

Genes investigated. Gene names, symbols and assay IDs are given. Asterisks (*) after gene names indicate control genes.

Supplementary table 2

Expression levels for the various genes investigated in by treatment group and area. ΔΔC_T_ values are given. As C_T_ values indicate number of replication cycles to exponential growth, lower C_T_ values indicate a higher original number of transcripts.

Supplementary table 3

Treatment effects of short-term treatment with *p*-CPA or paroxetine, corrected for multiple comparisons. Results of significance tests for global effects on a specific area (last row) and specific genes (last column) are given. Tests for a gene in a specific terminal area were only performed if a significant effect on the area in question had been obtained. The raphe region was analysed separately. Numbers indicate expression levels relative to the saline group, with asterisks (*) indicating level of significance for the comparison. * *p*<0.05, ** *p*<0.01

.

*Tables*

Supplementary table 1: Genes investigated

| **Gene name** | **Gene symbol** | **Assay ID** |
| --- | --- | --- |
| Hydroxytryptamine receptor 1A | *Htr1a* | Rn00561409_s1 |
| Hydroxytryptamine receptor 1B | *Htr1b* | Rn01637747_s1 |
| Hydroxytryptamine receptor 1D | *Htr1d* | Rn00563397_s1 |
| Hydroxytryptamine receptor 2A | *Htr2a* | Rn00568473_m1 |
| Hydroxytryptamine receptor 2C | *Htr2c* | Rn00562748_m1 |
| Hydroxytryptamine receptor 3A | *Htr3a* | Rn00667026_m1 |
| Hydroxytryptamine receptor 4 | *Htr4* | Rn00563402_m1 |
| Hydroxytryptamine receptor 6 | *Htr6* | Rn00577615_m1 |
| Hydroxytryptamine receptor 7 | *Htr7* | Rn00576048_m1 |
| Monoamine oxidase A | *Maoa* | Rn01430950_m1 |
| Monoamine oxidase B | *Maob* | Rn00566203_m1 |
| Tryptophan hydroxylase 2 | *Tph2* | Rn00598017_m1 |
| Vesicular monoamine transporter 2 | *Slc18a2* | Rn00564688_m1 |
| Serotonin transporter | *Slc6a4* | Rn00564737_m1 |
| Dopa-decarboxylase | *Ddc* | Rn00561113_m1 |
| FEV (ETS oncogene family) | *Fev* | Rn00462220_m1 |
| GATA binding protein 2 | *Gata2* | Rn00583735_m1 |
| Acheate-scute homolog 1 | *Ascl1* | Rn00574345_m1 |
| Brain-derived neurotrophic factor | *Bdnf* | Rn02531967_s1 |
| Neurotrophic tyrosine kinase, receptor, type 2 | *Ntrk2* | Rn01441749_m1 |
| S100 calcium binding protein A10 | S100a10 | Rn00821296_g1 |
| Fos proto-oncogene, AP-1 transcription subunit | *Fos* | Rn02396759_m1 |
| Hydroxymethylbilane synthase* | *Hmbs* | Rn00565886_m1 |
| Peptidylprolyl isomerase A (cyclophilin A)* | *Ppia* | Rn00690933_m1 |
| Glyceraldehyde 3-phosphate dehydrogenase* Tryptophan 5-monooxygenase activation protein Ζ* | *Gapdh*  *Yhwaz* | Rn01775763_g1 Rn00565886_m1 |
| 18s ribosomal RNA* | *18s* | Hs99999901_s1 |

Supplementary table 2: Gene expression effects of short-term manipulation of synaptic 5-HT levels (ΔΔC_t_ values)

|  | | |  |  |  |  |  |  |  |  |  |  |  |
| --- | --- | --- | --- | --- | --- | --- | --- | --- | --- | --- | --- | --- | --- |
|  |  | **Amygdala** | | **Hippocampus** | | **Striatum** | | **Hypothalamus** | | **PFC** |  | **Raphe** | |
| **Gene** | **Treatment** | **N** | **Means±S.E.** | **N** | **Means±S.E.** | **N** | **Means±S.E.** | **N** | **Means±S.E.** | **N** | **Means±S.E.** | **N** | **Means±S.E.** |
| *Htr1a* | p-CPA | 11 | 5.59±0.07 | 11 | 0.13±0.04 | 11 | 8.41±0.21 | 11 | 1.59±0.07 | 11 | 1.64±0.08 | 11 | 2.21±0.09 |
|  | Paroxetine | 11 | 5.47±0.09 | 11 | 0.04±0.04 | 11 | 8.39±0.22 | 11 | 1.63±0.05 | 10 | 1.68±0.05 | 11 | 2.17±0.07 |
|  | NaCl | 11 | 5.62±0.06 | 11 | 0.21±0.07 | 11 | 8.14±0.25 | 11 | 1.58±0.03 | 11 | 1.71±0.11 | 11 | 2.05±0.03 |
| *Htr1b* | p-CPA | 11 | 6.54±0.07 | 11 | 2.48±0.07 | 11 | 5.20±0.06 | 11 | 3.07±0.05 | 11 | 3.14±0.11 | 11 | 3.77±0.04 |
|  | Paroxetine | 11 | 6.76±0.04 | 11 | 2.66±0.09 | 11 | 5.30±0.08 | 11 | 3.12±0.06 | 10 | 3.40±0.06 | 11 | 3.72±0.04 |
|  | NaCl | 11 | 6.83±0.03 | 11 | 2.50±0.06 | 11 | 5.46±0.11 | 11 | 3.07±0.05 | 11 | 3.27±0.09 | 11 | 3.71±0.06 |
| *Htr1d* | p-CPA | 11 | 9.32±0.13 | 11 | 8.26±0.26 | 11 | 6.38±0.08 | 11 | 5.68±0.09 | 11 | 6.22±0.33 | 11 | 5.69±0.23 |
|  | Paroxetine | 11 | 9.54±0.10 | 11 | 7.71±0.26 | 11 | 6.40±0.05 | 11 | 5.43±0.17 | 10 | 6.27±0.19 | 11 | 5.69±0.10 |
|  | NaCl | 11 | 9.54±0.17 | 11 | 8.38±0.14 | 11 | 6.62±0.10 | 11 | 5.52±0.09 | 11 | 6.04±0.38 | 11 | 5.52±0.06 |
| *Htr2a* | p-CPA | 11 | 5.77±0.06 | 11 | 4.22±0.08 | 11 | 5.62±0.12 | 11 | 2.14±0.08 | 11 | 1.05±0.10 |  |  |
|  | Paroxetine | 11 | 6.05±0.04 | 11 | 4.44±0.09 | 11 | 6.10±0.11 | 11 | 2.51±0.06 | 10 | 1.19±0.08 |  |  |
|  | NaCl | 11 | 6.01±0.05 | 11 | 4.41±0.10 | 11 | 5.91±0.07 | 11 | 2.22±0.08 | 11 | 1.03±0.10 |  |  |
| *Htr2c* | p-CPA | 11 | 5.92±0.07 | 11 | 2.01±0.14 | 11 | 5.01±0.11 | 11 | 1.42±0.07 | 11 | 3.69±0.12 |  |  |
|  | Paroxetine | 11 | 5.64±0.13 | 11 | 2.18±0.21 | 11 | 5.11±0.08 | 11 | 1.34±0.05 | 10 | 3.84±0.07 |  |  |
|  | NaCl | 11 | 5.78±0.10 | 11 | 2.10±0.13 | 11 | 5.62±0.13 | 11 | 1.32±0.04 | 11 | 3.68±0.14 |  |  |
| *Htr3a* | p-CPA | 11 | 7.24±0.06 | 11 | 4.08±0.05 | 11 | 6.57±0.09 | 11 | 3.25±0.08 | 11 | 4.45±0.08 |  |  |
|  | Paroxetine | 11 | 7.27±0.05 | 11 | 4.24±0.09 | 11 | 6.50±0.06 | 11 | 3.49±0.06 | 10 | 4.40±0.09 |  |  |
|  | NaCl | 11 | 7.48±0.07 | 11 | 4.23±0.08 | 11 | 6.84±0.13 | 11 | 3.39±0.09 | 11 | 4.37±0.06 |  |  |
| *Htr4* | p-CPA | 11 | 8.45±0.06 | 11 | 3.23±0.08 | 11 | 7.03±0.08 | 11 | 3.63±0.05 | 11 | 6.86±0.10 |  |  |
|  | Paroxetine | 11 | 8.34±0.08 | 11 | 3.31±0.08 | 11 | 7.02±0.09 | 11 | 3.54±0.04 | 10 | 6.80±0.12 |  |  |
|  | NaCl | 11 | 8.61±0.04 | 11 | 3.41±0.08 | 11 | 7.15±0.09 | 11 | 3.53±0.02 | 11 | 6.93±0.12 |  |  |
| *Htr6* | p-CPA | 11 | 9.35±0.09 | 11 | 5.60±0.08 | 11 | 6.84±0.08 | 11 | 6.35±0.07 | 11 | 4.66±0.07 |  |  |
|  | Paroxetine | 11 | 9.3±0.09 | 11 | 5.41±0.12 | 11 | 6.75±0.07 | 11 | 6.25±0.07 | 10 | 4.26±0.07 |  |  |
|  | NaCl | 11 | 9.35±0.10 | 11 | 5.35±0.09 | 11 | 6.85±0.08 | 11 | 6.31±0.05 | 11 | 4.16±0.04 |  |  |
| *Htr7* | p-CPA | 11 | 8.68±0.06 | 11 | 5.12±0.04 | 11 | 11.03±0.11 | 11 | 3.68±0.06 | 11 | 5.95±0.07 |  |  |
|  | Paroxetine | 11 | 8.65±0.07 | 11 | 5.20±0.10 | 11 | 10.99±0.13 | 11 | 3.68±0.02 | 10 | 6.05±0.05 |  |  |
|  | NaCl | 11 | 8.79±0.04 | 11 | 5.14±0.06 | 11 | 10.98±0.12 | 11 | 3.66±0.03 | 11 | 5.95±0.06 |  |  |
| *Maoa* | p-CPA | 11 | 6.3±0.03 | 11 | 3.11±0.04 | 11 | 6.24±0.05 | 11 | 1.75±0.06 | 11 | 2.52±0.07 | 11 | 2.20±0.06 |
|  | Paroxetine | 11 | 6.07±0.07 | 11 | 2.97±0.05 | 11 | 6.11±0.07 | 11 | 1.68±0.03 | 10 | 2.31±0.07 | 11 | 2.00±0.08 |
|  | NaCl | 11 | 6.25±0.04 | 11 | 3.14±0.07 | 11 | 6.26±0.05 | 11 | 1.63±0.03 | 11 | 2.36±0.10 | 11 | 2.06±0.06 |
| *Maob* | p-CPA | 11 | 4.59±0.07 | 11 | 1.69±0.07 | 11 | 4.04±0.06 | 11 | -0.51±0.13 | 11 | 0.67±0.10 | 11 | 1.27±0.05 |
|  | Paroxetine | 11 | 4.46±0.08 | 11 | 1.61±0.05 | 11 | 4.05±0.07 | 11 | -0.30±0.08 | 10 | 0.35±0.14 | 11 | 1.51±0.09 |
|  | NaCl | 11 | 4.76±0.07 | 11 | 1.80±0.04 | 11 | 4.29±0.09 | 11 | -0.32±0.06 | 11 | 0.65±0.07 | 11 | 1.45±0.05 |
| *Bdnf* | p-CPA | 11 | 7.87±0.10 | 11 | 4.01±0.08 | 11 | 9.75±0.33 | 11 | 4.51±0.14 | 11 | 3.52±0.18 | 11 | 5.12±0.19 |
|  | Paroxetine | 11 | 8.36±0.08 | 11 | 4.51±0.06 | 11 | 10.15±0.30 | 11 | 4.86±0.11 | 10 | 3.85±0.07 | 11 | 6.17±0.19 |
|  | NaCl | 11 | 8.17±0.06 | 11 | 4.56±0.07 | 11 | 9.82±0.029 | 11 | 4.69±0.06 | 11 | 3.58±0.16 | 11 | 5.94±0.14 |
| *Ntrk2* | p-CPA | 11 | 1.49±0.05 | 11 | -1.75±0.06 | 11 | 1.71±0.05 | 11 | -2.14±0.05 | 11 | -2.04±0.08 | 11 | -0.90±0.14 |
|  | Paroxetine | 11 | 1.53±0.07 | 11 | -1.62±0.06 | 11 | 1.78±0.03 | 11 | -2.13±0.05 | 10 | -2.23±0.12 | 11 | -0.87±0.06 |
|  | NaCl | 11 | 1.59±0.04 | 11 | -1.61±0.08 | 11 | 1.89±0.04 | 11 | -2.12±0.06 | 11 | -2.16±0.05 | 11 | -0.97±0.07 |
| *S100a10* | p-CPA | 11 | 5.87±0.11 | 11 | 2.41±0.03 | 11 | 6.72±0.14 | 11 | 1.42±0.10 | 11 | 1.48±0.09 | 11 | 0.71±0.10 |
|  | Paroxetine | 11 | 5.83±0.10 | 11 | 2.40±0.06 | 11 | 6.62±0.10 | 11 | 1.58±0.07 | 10 | 1.53±0.09 | 11 | 0.69±0.06 |
|  | NaCl | 11 | 6.02±0.07 | 11 | 2.52±0.03 | 11 | 6.39±0.16 | 11 | 1.49±0.11 | 11 | 1.64±0.07 | 11 | 0.66±0.07 |
| *Slc6a4* | p-CPA | 9 | 14.38±0.20 | 11 | 12.49±0.20 | 10 | 15.36±0.18 | 11 | 10.08±0.19 | 11 | 10.43±0.10 | 11 | 2.90±0.23 |
|  | Paroxetine | 11 | 14.11±0.19 | 10 | 11.91±0.17 | 11 | 14.44±0.13 | 11 | 9.50±0.22 | 10 | 9.72±0.23 | 11 | 2.78±0.24 |
|  | NaCl | 10 | 14.23±0.19 | 10 | 12.26±0.19 | 10 | 14.88±0.15 | 11 | 9.11±0.32 | 11 | 9.81±0.22 | 11 | 2.27±0.13 |
| *Tph2* |  |  |  |  |  |  |  |  |  |  |  | 11 | 1.91±0.59 |
|  |  |  |  |  |  |  |  |  |  |  |  | 11 | 2.46±0.71 |
|  |  |  |  |  |  |  |  |  |  |  |  | 11 | 1.62±0.22 |
| *Ddc* |  |  |  |  |  |  |  |  |  |  |  | 11 | 2.38±0.10 |
|  |  |  |  |  |  |  |  |  |  |  |  | 11 | 2.42±0.11 |
|  |  |  |  |  |  |  |  |  |  |  |  | 11 | 2.06±0.06 |
| *Slc18a2* |  |  |  |  |  |  |  |  |  |  |  | 11 | 1.65±0.12 |
|  |  |  |  |  |  |  |  |  |  |  |  | 11 | 1.78±0.10 |
|  |  |  |  |  |  |  |  |  |  |  |  | 11 | 1.54±0.11 |
| *fev* |  |  |  |  |  |  |  |  |  |  |  | 11 | 5.15±0.17 |
|  |  |  |  |  |  |  |  |  |  |  |  | 11 | 5.37±0.24 |
|  |  |  |  |  |  |  |  |  |  |  |  | 11 | 4.76±0.10 |
| *Gata2* |  |  |  |  |  |  |  |  |  |  |  | 11 | 4.92±0.24 |
|  |  |  |  |  |  |  |  |  |  |  |  | 11 | 4.79±0.10 |
|  |  |  |  |  |  |  |  |  |  |  |  | 11 | 4.66±0.06 |
| *Ascl1* |  |  |  |  |  |  |  |  |  |  |  | 11 | 4.37±0.07 |
|  |  |  |  |  |  |  |  |  |  |  |  | 11 | 4.04±0.10 |
|  |  |  |  |  |  |  |  |  |  |  |  | 11 | 4.18±0.08 |
| *Fos* | p-CPA | 11 | 7.02±0.15 | 11 | 3.78±0.11 | 11 | 7.40±0.13 | 11 | 3.00±0.17 | 11 | 3.42±0.19 | 11 | 3.38±0.13 |
|  | Paroxetine | 11 | 6.96±0.14 | 11 | 4.12±0.25 | 11 | 6.86±0.21 | 11 | 2.92±0.16 | 10 | 2.72±0.21 | 11 | 3.74±0.11 |
|  | NaCl | 11 | 7.26±0.14 | 11 | 4.43±0.12 | 11 | 7.48±0.25 | 11 | 3.34±0.09 | 11 | 3.10±0.21 | 11 | 3.91±0.08 |
| S.E.=standard error of the mean | | | |  |  |  |  |  |  |  |  |  |  |

Supplementary table 3: Gene expression effects of short-term manipulation of synaptic 5-HT levels (corrected by way of permutation analysis)

|  |  |  |  |  |  |  |  |  |  |  |  |  |  |  |
| --- | --- | --- | --- | --- | --- | --- | --- | --- | --- | --- | --- | --- | --- | --- |
|  | **Amygdala** | | **Hippocampus** | | **Striatum** |  | **Hypothalamus** | | **Prefrontal cortex** | | **Raphe** |  | **Gene signal: *p-CPA*** | **Gene signal: paroxetine** |
|  | *p*-CPA | SSRI | *p*-CPA | SSRI | *p*-CPA | SSRI | *p*-CPA | SSRI | *p*-CPA | SSRI | *p*-CPA | SSRI |  |  |
| *Htr1a* | 1.03 | 1.11 | 1.06 | 1.12 | 0.83 | 0.83 | 0.99 | 0.96 | 1.05 | 1.18 | 0.89 | 0.92 |  |  |
| *Htr1b* | 1.20 ** | 1.05 | 1.01 | 0.89 | 1.20 | 1.11 | 1.09 | 0.91 | 1.09 | 1.09 | 0.69 | 0.99 | * |  |
| *Htr1d* | 1.17 | 1.00 | 1.09 | 1.59 | 1.18 | 1.16 | 0.90 | 1.06 | 0.88 | 1.25 | 0.89 | 0.89 |  |  |
| *Htr2a* | 1.18 * | 0.96 | 1.15 | 0.98 | 1.22 | 0.87 | 1.05 | 0.81 | 0.99 | 0.95 |  |  |  |  |
| *Htr2c* | 0.91 | 1.10 | 1.06 | 0.94 | 1.53 * | 1.43 | 0.93 | 0.98 | 1.00 | 0.86 |  |  |  |  |
| *Htr3a* | 1.18 | 1.15 | 1.11 | 0.99 | 1.21 | 1.26 | 1.10 | 0.92 | 0.95 | 1.02 |  |  |  |  |
| *Htr4* | 1.12 | 1.20 | 1.14 | 1.20 | 1.09 | 1.10 | 0.93 | 0.99 | 1.05 | 1.09 |  |  |  |  |
| *Htr6* | 1.00 | 1.03 | 0.84 | 1.03 | 1.01 | 1.07 | 0.97 | 1.04 | 0.71 *** | 0.92 |  |  | ** |  |
| *Htr7* | 1.08 | 1.10 | 1.08 | 1.10 | 0.97 | 0.99 | 0.99 | 0.98 | 1.00 | 0.93 |  |  |  |  |
| *Maoa* | 0.96 | 1.13 | 0.96 | 1.12 | 1.02 | 1.11 | 0.92 | 0.96 | 0.90 | 0.99 | 0.90 | 1.04 |  |  |
| *Maob* | 1.13 | 1.23 | 1.13 | 1.14 | 1.19 | 1.18 | 1.14 | 0.98 | 0.99 | 1.18 | 1.13 | 0.96 |  |  |
| *Bdnf* | 1.23 | 0.87 | 1.46 ** | 1.03 | 1.05 | 0.79 | 1.13 | 0.88 | 1.04 | 0.79 | 1.77 ** | 0.85 | ** |  |
| *Ntrk2* | 1.08 | 1.04 | 1.11 | 1.00 | 1.13 | 1.07 | 1.01 | 1.00 | 0.92 | 1.07 | 0.95 | 0.94 |  |  |
| *S100a10* | 1.11 | 1.13 | 1.08 | 1.09 | 0.79 | 0.84 | 1.06 | 0.94 | 1.12 | 0.84 | 0.97 | 0.98 |  |  |
| *Slc6a4* | 0.90 | 1.08 | 0.90 | 1.27 | 0.72 | 1.35 | 0.51 | 0.76 | 0.65 * | 1.25 | 0.65 * | 0.71 |  |  |
| *Tph2* |  |  |  |  |  |  |  |  |  |  | 0.82 | 0.56 ** | N/A | N/A |
| *Ddc* |  |  |  |  |  |  |  |  |  |  | 0.80 ** | 0.78 | N/A | N/A |
| *Slc18a2* |  |  |  |  |  |  |  |  |  |  | 0.93 | 0.84 | N/A | N/A |
| *Fev* |  |  |  |  |  |  |  |  |  |  | 0.76 (p=.07) | 0.66 | N/A | N/A |
| *Gata2* |  |  |  |  |  |  |  |  |  |  | 0.84 | 0.91 | N/A | N/A |
| *Ascl1* |  |  |  |  |  |  |  |  |  |  | 0.87 (p=.07) | 1.10 | N/A | N/A |
| **Area signal: *p*-CPA** | * |  | ** |  | * |  |  |  | ** |  |  |  |  |  |
| **Area signal: paroxetine** | |  |  |  |  |  |  |  |  |  |  |  |  |  |
| n=10-11 (Slc6a4 amygdala=9) | |  |  |  |  |  |  |  |  |  |  |  |  |  |
